# Truncated Estimation of Skating Force-Velocity Profiling When Using High-Speed Video-Based Methods Compared to Radar-Derived Processing

**DOI:** 10.3389/fbioe.2021.661744

**Published:** 2021-06-24

**Authors:** Jerome Perez, Gaël Guilhem, Franck Brocherie

**Affiliations:** ^1^French Institute of Sport (INSEP), Laboratory Sport, Expertise and Performance (EA 7370), Paris, France; ^2^French Ice Hockey Federation, Cergy, France

**Keywords:** biomechanics, ice hockey, assessment, muscle capacities, sprint performance

## Abstract

This study aimed to compare the force-velocity mechanical variables derived from high-speed video- and radar-based method during forward skating sprint in ice hockey. Thirteen elite female ice hockey players performed two 40-m forward skating sprints to determine, in the horizontal plane, maximal velocity reached (V_max_), relative maximal theoretical force (F_0_), maximal theoretical velocity (V_0_), relative maximal power (P_max_), linear slope of the force-velocity relationship (FV slope), maximal value of the ratio of force (RF_max_) and index of force application technique (Drf). Two different high-speed video-based methods adding a time shift (ST-TS) or not (ST) were used and independently compared to the radar-derived method. ST and ST-TS showed significant mean differences (all *p* < 0.002) compared to radar-derived processing for all variables except for V_0_ (*p* = 0.26) and V_max_ (*p* = 0.13) inferred from ST. In reference to radar-derived variables, ST-TS significantly induced *larger* lower values compared to radar of the main forward skating sprint determinants (P_max_, F_0_, RF_max_ and Drf) and *moderate-to-large* overestimation for velocity variables (V_0_ and V_max_). Correlations between ST or ST-TS and radar-derived methods ranged from *trivial* for velocity variables to *very large* for force and power variables. Consequently, practitioners must be aware that using such high-speed video-based methods would permit to determine mechanical variables at the cost of much lower accuracy and reliability than the radar-derived method.

## Introduction

Horizontal force, power output and acceleration achieved during forward skating sprint are key determinants of ice hockey performance (Pearsall et al., 2013; Perez et al., 2020). Recently, the application of the force-velocity (FV) profiling during forward skating has been proposed using either *in situ* radar-derived (Perez et al., 2019, 2020) or high-speed video-based (Stenroth et al., 2020) measurements with potential practical applications for training individualization and monitoring (Morin and Samozino, 2016; Jimenez-Reyes et al., 2019, 2020).

Unfortunately, on-ice conditions do not allow the use of gold standard methods (i.e., force plates) to clearly determine concurrent validity for both methods. Several studies have reported acceptable validity of running sprint-related velocity-time curve inferred from radar device (Simperingham et al., 2016) and its derived mechanical variables (Samozino et al., 2016). Potential limitations (i.e., change in sprinting posture during the first few steps) that could affect measurements validity (Bezodis et al., 2012; Haugen and Buchheit, 2016) has been successfully corrected by shifting the measures by a 0.3 s time delay to not overestimate force and power measurements (Samozino, 2018). As previously mentioned, this method has been successfully applied to skating sprints (Perez et al., 2019). While one may argue that such method requires specific data processing, it allows practitioners to accurately assess mechanical determinants of forward skating sprint such as relative maximal theoretical horizontal force (F_0_), power (P_max_) and the maximal ratio of the horizontal component of the ground-reaction force to the corresponding resultant force (RF_max_) (Perez et al., 2020). Recently, Stenroth et al. (2020) adapted an alternative method based on the measurement of split time (ST method) with high-speed video (i.e., 240 frames per second) primarily developed to assess FV profiling during running sprint (Samozino et al., 2016). Romero-Franco et al. (2017) demonstrated that, in running sprint condition, high-speed video-based method was valid [*r* = 0.974–0.999, *p* < 0.001, intra-class correlation coefficient (ICC) = 0.987–1.00] and reliable (coefficient of variation = 0.14%) to determinate FV mechanical variables compared to those derived from the radar method. In the context of ice skating, Stenroth et al. (2020) proposed to add a time shift (ST-TS method) in order to easily determine on-ice forward skating sprint mechanical variables. On the one hand, this approach uses low-cost handling tools more accessible to practitioners. On the other hand, Stenroth et al. (2020) reported substantial bias when skating sprint mechanical variables are inferred from this alternative procedure—especially during the acceleration phase (F_0_, P_max_ and RF_max_)—compared to the data obtained using a continuous video tracking. For instance, inter-trial reliability, determined with ICCs, of high-speed video-based method appears to be lower to those reported using radar-derived method for maximal theoretical velocity (V_0_; ICCs ranging from 0.198 to 0.343 vs. 0.86, respectively) and maximal velocity (V_max_) reached during skating sprint (ICCs ranging from 0.497 to 0.596 vs. 0.91, respectively) (Perez et al., 2019). In addition, while Stenroth et al. (2020) suggested to analyze only the fastest trial, several running- and skating-based studies showed that averaging sprint trials improve the reliability (Perez et al., 2019; Simperingham et al., 2019).

This study therefore aimed to compare the force-velocity mechanical variables derived from high-speed video- and radar-based method during on-ice forward skating sprint in ice hockey. In line with the results from Stenroth et al. (2020) showing significant differences between two different video-based methods (ST-TS and continuous tracking), we hypothesized that significant differences would be found between the high-speed video-based methods and the radar-derived method, the latter remaining the preferred approach to infer FV-related mechanical variables.

## Methods

### Participants

Thirteen elite female ice hockey players of the French national team (mean ± SD: age, 21.0 ± 3.2 years; height, 1.65 ± 0.10 m; body mass, 64.8 ± 10.1 kg; playing experience, 14.3 ± 3.1 years) participated in this study. They were free from any musculoskeletal injury of the lower limb during the 3 months preceding data collection. All participants received a clear explanation of the experimental procedure before they provided written consent to participate. The protocol was integrated in the regular training of the players and was approved by ethics committee Ouest IV.

### Experimental Design

Forward skating sprint FV profile was determined during the pre-season training camp of the national team as described elsewhere (Perez et al., 2020). Players were instructed to wear their full ice hockey equipment and were weighed (72.6 ± 9.4 kg fully equipped) before the testing session. The study compared mechanical variables determined using two high-speed video-based methods (ST and ST-TS) (Romero-Franco et al., 2017; Stenroth et al., 2020) and those obtained using the radar-derived method (Perez et al., 2019) for the same forward skating sprint for each player. ST method has been validated by Romero-Franco et al. (2017) during running sprint and designed into a spreadsheet by Morin and Samozino (2019). ST-TS method was recently developed by Stenroth et al. (2020) which added an optimized parameter in the context of forward skating sprint, named time shift, to the original method (Romero-Franco et al., 2017). This ST-TS approach aimed to remove uncertainty in identifying the onset of horizontal force generation by changing the duration of the first-time interval while maintaining the differences between other split times. On the ice, although skate’s blade must be oriented perpendicularly to the intended direction of motion inducing a medio-lateral force, the capacity to generate an efficient F_0_ seems paramount for an efficient forward skating performance (Pearsall et al., 2013; Perez et al., 2020).

### Testing Procedures

Players had a general warm-up of 15 min including skating skills and three progressive 40-m forward skating sprints at self-perceived increasing skating velocity, as previously detailed (Perez et al., 2019). Then, players performed two on-ice (∼10°C ambient temperature and ∼75% relative humidity) 40-m maximal forward skating sprints interspersed by 4 min of passive rest. Players started from a standing straight position with skates’ blades positioned in a “V” stance and skated as fast as possible while holding their stick, mimicking in-game skating. Trials were assessed by recording each sprint using a radar device (Stalker ATS II; Applied Concepts, Plano, TX, United States) and a high-speed camera (iPhone 6, Apple Inc., United States, framerate 240 frames per second, resolution 1,280 × 720 pixels). Instantaneous horizontal velocity (V_h_, in m.s^–1^) was measured by the radar device at a 47 Hz sampling frequency. The radar was located 3.5 m behind the starting line at a height of 1 m from the ice surface, corresponding approximatively to the average height of players’ center of mass (Samozino, 2018; Perez et al., 2019, 2020). Meanwhile, to record the video of each sprint, the camera was held by the same practitioner at a height of 1.5 m (in the frontal plane) and positioned at 20 m from the starting line and at 11.5 m from the skating line (Samozino, 2018). The practitioner moved the camera around itself in order to film the sprint from the side and register the entire sprint. According to Romero-Franco et al. (2017), and using Thales theorem, video parallax was corrected to ensure that 5-, 10-, 15-, 20-, 25-, 30-, 35-, and 40-m split times were measured properly. The correction of the parallax was done by positioning the different markers (i.e., vertical markers) not exactly at the associated distances (i.e., 5, 10, 15, 20, 25, 30, 35, and 40 m from the starting line), but at adjusted positions so that the players were filmed to cross the markers with their hip when they were exactly at these targeted distances (i.e., to correct for the parallax error, the markers positions were, respectively, positioned at 6.96, 11.30, 15.65, 20.00, 24.35, 28.70, 33.04, and 37.39 m from the start line). Skating line was parallel to the line of the markers with a 1.5 m average separation between the lines.

### Data Processing

**FIGURE 1:**
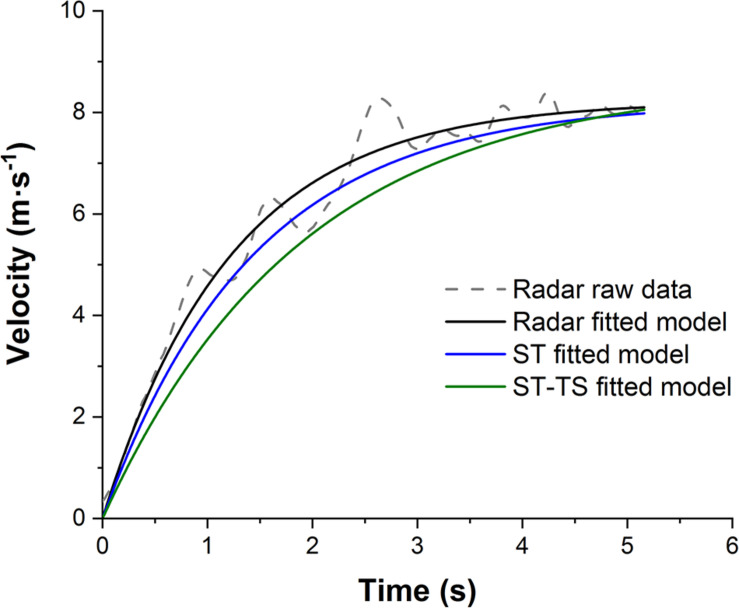
Raw data (dotted line) of the velocity-time curve measured by radar and fitted model (straight lines) derived from each method (high-speed video split time (ST) in blue, high-speed video split time with time shift (ST-TS) in green and radar in black). Data were collected from the same skating sprint of one player.

Radar raw data acquisitions were saved on Stalker^TM^ ATS System software (Version 5.0.3.0, Applied Concepts, Inc., Texas, United States) and imported to a custom-made Origin script (Version 8.0, OriginLab Corporation, United States) to model mechanical variables from the V_h_-time curve data set (Figure 1; Perez et al., 2019). F_0_ (in N.kg^–1^), V_0_ (in m.s^–1^), relative maximal horizontal power P_max_ (in W.kg^–1^), slope of the linear relationship between force expressed relatively to body mass and velocity (FV slope, in N.m.kg^–1^.s^–1^), maximal ratio of force (RF_max_, in %) and index of force application technique (Drf, in %) were determined.

Considering the ST and ST-TS methods, only one rater performed the analysis. Kinovea software (Version 0.8.26, 2017) was used to measure the split times by manually selecting the frames in which the players passed the markers with their hip. Spreadsheets designed by Morin and Samozino (2019) and Stenroth et al. (2020) were used to calculate FV profile and sprint mechanical variables using ST and ST-TS methods, respectively. Position of the player was modeled as a function of time with the equation (1) for ST (Morin and Samozino, 2019) and equation (2) for ST-TS adding the time shift parameter *c* (Stenroth et al., 2020).

(1) $$x\left(t\right)=v_{max}\times \left(t+\tau e^{-t/\tau }\right)-v_{max}\times \tau$$

(2) $$x\left(t\right)=v_{max}\times \left(t+c+\tau e^{-\left(tc\right)/\tau }\right)-v_{max}\times \tau$$

Constant maximal velocity (V_max_, plateau of the velocity) and τ (acceleration time constant) were found using built-in solver function of Excel (Microsoft Corporation, Redmond, Washington, United States) (Figure 1). The solver was set to minimize the sum of squared differences between the modeled and actual positions of the player by altering the constants. A non-linear generalized reduced gradient algorithm was used as the solving method. After estimating V_max_ and τ, all mechanical variables from the FV relationship could be modeled after integration:

(3) $$V_{h}\left(t\right)=V_{max}\times \left(1-e^{-\frac{t}{\tau }}\right)$$

(4) $$F_{h}\left(t\right)=m\times a_{h}\left(t\right)+F_{aero}$$

where F_h_ (in N) is the net horizontal antero-posterior of the ground reaction force, m (in kg) is the system mass which included full ice hockey equipment during the on-ice measures and F_aero_ (in N) is the resistance due to aerodynamic friction force, individualized for the participants (i.e., depending on the height and body mass equipped). Mean net horizontal antero-posterior power output (P_h_, in W) was then modeled at each instant as the product of F_h_ and V_h_:

(5) $$P_{h}\left(t\right)=F_{h}\left(t\right)\times V_{h}\left(t\right)$$

In order to synchronize the two devices, the start of the skating sprint was determined as the moment in which there is the first movement of the player detected by visual inspection with high-speed video and the center-of-mass velocity above an arbitrary speed of 0.2 m.s^–1^ for the radar (Romero-Franco et al., 2017). A total of 21 skating sprints were analysed individually.

### Statistical Analysis

All data were analyzed using custom written scripts (Origin 2020, OriginLab Corporation, Northampton, MA) and expressed as mean ± standard deviation (SD). Statistical significance was set at *p* < 0.05. Normality was confirmed using the Shapiro-Wilk test. To investigate systematic bias (mean differences), a paired sample *t*-test was conducted between the same mechanical variables inferred from ST, ST-TS and radar-derived methods. The level of concordance between ST, ST-TS and radar-derived methods was estimated by the Bland and Altman plots (Bland and Altman, 2010) with a 95% limit of agreements (95% LoA, mean bias; mean difference, ±1.96 SD). The Cohen’s *d* scale was used to interpret the effect sizes (Hopkins et al., 2009). To determine the inter-method relative reliability of computed variables, ICCs were calculated (Hopkins et al., 2009). Pearson’s product-moment correlation analysis was used to determine the relationship between the same mechanical variables derived from ST, ST-TS and radar-derived methods (Hopkins et al., 2009). The typical error of the estimate (TEE) (95% confidence intervals, 95% CI) was calculated and standardized for the purpose of interpretation (Hopkins, 2000).

## Results

**FIGURE 2:**
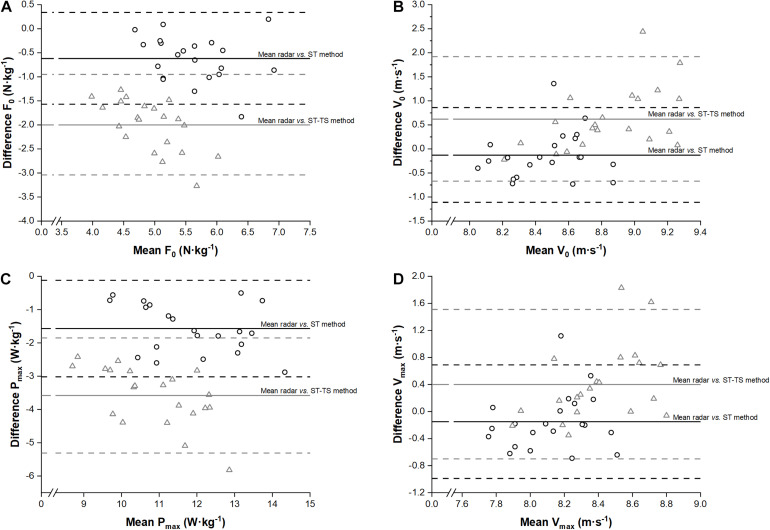
Bland and Altman plots of both high-speed video-based methods and radar-derived method for F_0_ (panel **(A)**, V_0_ (panel **(B)**, P_max_ (panel **(C)** and V_max_ (panel **(D)**. Dark circles and lines represented differences between high-speed video split time method (ST) and radar-derived method while gray triangles and lines represented differences between high-speed video split time with time shift method (ST-TS) and radar-derived method. Upper and lower horizontal dotted lines represent the 95% limits of agreement (mean ± 1.96 SD of the difference between methods).

The mean time shift parameter (*c*) of the ST-TS method for all the player was 0.268 ± 0.053 s. ST and ST-TS showed significant mean differences compared to radar for all variables except for V_0_ (*p* = 0.26) and V_max_ (*p* = 0.13) inferred from ST (Table 1). For F_0_, V_0_, P_max_ and V_max_, the mean bias between ST, ST-TS and radar-derived methods were displayed as Bland and Altman plots (Figure 2). Bias and random errors for the other variables were reported in Tables 1, 2. ST showed a negative *small-to-moderate* difference compared to radar for all variables except for FV slope (positive *moderate* difference) (Table 1). ST-TS showed even *largely* lower values for F_0_, P_max_, RF_max_, and Drf variables and *moderate-to-large* higher values for V_0_, FV slope and V_max_ variables compared to radar (Table 2). Relative reliability (ICC) was *moderate-to-high* for F_0_, P_max_, and RF_max_ for both ST and ST-TS while other variables were *very low-to-low* (Tables 1, 2). Correlations between ST or ST-TS and radar ranged from *trivial* for V_max_ and V_0_ to *very large* for F_0_ (only for ST-TS), P_max_ and RF_max_ (Tables 1, 2). TEE was *small* for P_max_ to *very large* for V_0_, Drf and V_max_ for ST (Table 1) and ranged from *moderate* for F_0_, P_max_ and RF_max_ to *very large* for all other variables for ST-TS (Table 2).

**TABLE 1:** Differences in forward skating sprint mechanical variables determined with split time (ST) high-speed video-based method in reference to radar-based method.

	*p-*value	Mean difference (%)	±95% LOA	*d*; effect	ICC	*r*	*p-*value	TEE
F_0_ (N⋅kg^–1^)	<0.001	–0.62 (–10.88)	0.96	–0.85; *“moderate”*	0.74 (0.45–0.88) *“moderate”*	0.74 (0.46–0.89)	<0.001	0.90 (0.51–1.94) *“moderate”*
V_0_ (m⋅s^–1^)	0.264	–0.13 (–1.54)	0.99	–0.37; *“small”*	–0.10 (–0.54–0.35) *“very low”*	–0.04 (–0.47–0.40)	0.863	23.90 (15.87–30.85) *“very large”*
P_max_ (W⋅kg^–1^)	<0.001	–1.57 (–13.15)	1.45	–0.98; *“moderate”*	0.87 (0.70–0.94) *“high”*	0.88 (0.71–0.95)	<0.001	0.55 (0.33–0.98) *“small”*
FV slope (N⋅s⋅kg^–1⋅^m^–1^)	0.002	0.06 (9.32)	0.17	0.74; *“moderate”*	0.46 (0.02–0.74) *“low”*	0.49 (0.07–0.76)	0.027	1.79 (0.86–14.30) *“large”*
RF_max_ (%)	<0.001	–2.52 (–6.73)	2.45	–0.98; *“moderate”*	0.86 (0.67–0.94) *“high”*	0.85 (0.67–0.94)	<0.001	0.61 (0.37–1.12) *“moderate”*
Drf (%)	0.002	0.63 (9.62)	1.59	0.81; *“moderate”*	0.34 (–0.12–0.67) *“low”*	0.39 (–0.05–0.70)	0.082	2.38 (1.02–18.61) *“very large”*
V_max_ (m⋅s^–1^)	0.128	–0.15 (–1.86)	0.84	–0.47; *“small”*	0.03 (–0.43–0.45) *“low”*	0.08 (–0.37–0.49)	0.741	12.97 (6.24–18.63) *“very large”*

*LOA, limit of agreement (1.96^∗^SD); d, Cohen’ d effect size; ICC, intra class correlation coefficient (lower – upper limit); r, Pearson’s product–moment correlation; TEE, standardiszd typical error of the estimate.*

**TABLE 2:** Differences in forward skating sprint mechanical variables determined with split time with a time-shift (ST-TS) high-speed video-based method in reference to radar-based method.

	*p-*value	Mean difference (%)	±95% LOA	*d*; effect	ICC	*r*	*p-*value	TEE
F_0_ (N⋅kg^–1^)	<0.001	–2.00 (–40.27)	1.05	–1.72; *“large”*	0.58 (0.19–0.80) *“moderate”*	0.68 (0.35–0.86)	<0.001	1.07 (0.59–2.64) *“moderate”*
V_0_ (m⋅s^–1^)	<0.001	0.62 (6.92)	1.30	1.13; *“moderate”*	–0.01 (–0.47–0.42) *“very low”*	–0.05 (–0.48–0.39)	0.813	18.24 (14.42–22.71) *“very large”*
P_max_ (W⋅kg^–1^)	<0.001	–3.58 (–32.86)	1.73	–1.62; *“large”*	0.78 (0.52–0.90) *“high”*	0.84 (0.63–0.93)	<0.001	0.66 (0.39–1.23) *“moderate”*
FV slope (N⋅s⋅kg^–1⋅^m^–1^)	<0.001	0.27 (46.70)	0.18	1.73; *“large”*	0.23 (–0.24–0.60) *“low”*	0.29 (–0.17–0.64)	0.207	3.35 (1.20–5.93) *“very large”*
RF_max_ (%)	<0.001	–8.14 (–23.56)	3.12	–1.73; *“large”*	0.76 (0.49–0.90) *“high”*	0.76 (0.49–0.90)	<0.001	0.86 (0.49–1.80) *“moderate”*
Drf (%)	<0.001	2.48 (45.79)	1.70	–1.74; *“large”*	0.18 (–0.29–0.56) *“low”*	0.24 (–0.22–0.61)	0.313	4.14 (1.31–4.46) *“very large”*
V_max_ (m⋅s^–1^)	0.004	0.40 (4.71)	1.11	0.93; *“moderate”*	–0.06 (–0.51–0.38) *“low”*	–0.08 (–0.50–0.36)	0.722	12.00 (6.10–18.21) *“very large”*

*LOA, limit of agreement (1.96^∗^SD); d, Cohen’ d effect size; ICC, intra class correlation coefficient (lower – upper limit); r, Pearson’s product–moment correlation; TEE, standardized typical error of the estimate.*

## Discussion

This present study demonstrated that, comparatively to radar-derived processing, both high-speed video-based methods underestimated the force and power variables (i.e., F_0_, P_max_, RF_max_, and Drf, *small-*to-*large* differences), while velocity (i.e., V_0_ and V_max_) and FV slope variables were *moderately* to-*largely* overestimated by ST-TS method only. Furthermore, although mechanical variables derived from ST appeared to be more accurate than ST-TS, high-speed video-based methods seemed to be less accurate with *small*-to-*large* mean differences depending on variables compared to the measures obtained from the radar.

In line with Stenroth et al. (2020), our findings showed that the implementation of time shift in velocity data processing (ST-TS) induced a significant (*p* < 0.001) and *large* (*d* ranging from 1.62 to 1.74) underestimation (from –24 to–46% on average) of the main determinants of forward skating sprint performance (i.e., F_0_, P_max_, RF_max_, and Drf variables) when compared to radar-derived data processing (Perez et al., 2020). While significant differences between ST and radar (*p* ≤ 0.002) were also observed with a *moderate* effect size (*d* ranging 0.81–0.98), using original ST (Romero-Franco et al., 2017) appeared to reduce, but not fully remove, the underestimation of the main determinants of forward skating sprint performance (from–7 to–13%). Additionally, ST tended to improve the ICCs compared to ST-TS, especially for F_0_, P_max_, RF_max_, and Drf variables (0.34–0.87 vs. 0.18–0.78). Finally, our results showed that both F_0_, P_max_ and RF_max_ high-speed video-derived measures showed significant *large*-to*-very large* correlations (*r* ranging from 0.68 to 0.88) with those derived from the radar-derived method. However, these results (large range and *r* < 0.9) do not allow to clearly consider these methods as accurate as the reference radar-based method.

In their study, Stenroth et al. (2020) measured sprint skating velocity over a 30-m. Budarick et al. (2018) showed that this distance may not allow ice hockey players to reach their ultimate maximum velocity as acceleration was still positive at 34 m. The achievement of V_max_ is a prerequisite to reliably build the FV relationship during forward skating sprint inferred from the mono-exponential modeling of the velocity-time curve (Samozino et al., 2016; Perez et al., 2019). It is therefore unlikely that a 30-m distance may be consistently long enough for ice hockey players to reach their V_max_ (Perez et al., 2020). This setup could thus induce higher variability in measured peak velocity compared to longer sprint distance and partly explain the difference in V_max_ measurement. In line with the findings of Stenroth et al. (2020), we observed a significant overestimation of V_0_ and V_max_ assessed over 40 m using ST-TS compared to radar, leading to higher differences in FV slope. This overestimation could be mainly attributed to the added time shift parameter, which has been shown to lower the curvature of the velocity-time relationship (Stenroth et al., 2020). Considering ST, V_0_, and V_max_ were not significantly different compared to the radar-derived measures with a *small* difference (*d* ranging 0.37 and 0.47, respectively). Importantly, V_0_ and V_max_ variables inferred from both high-speed video-based methods showed *very large* TEEs (12.00–23.90), *very low* ICCs (0.01–0.10) and no correlations (*r* ranging from 0.04 to 0.08) compared to the same radar-derived variables. These results confirm that the assessment of instantaneous metric (V_max_) or extracted from the FV profile requires continuous measures with sufficient sampling frequency as allowed using the radar.

While the current findings revealed *large*-to*-very large* correlations between high-speed video-based and radar-derived method, particularly for mechanical determinants of forward skating sprint, high-speed video-based methods present some limitations for measuring mechanical variables. The accuracy of the determination of the frame corresponding to the start of the sprint which corresponds to the beginning of the force production is indeed a crucial factor for accurate assessment of sprint mechanical variable (Samozino, 2018). Unfortunately, this standardized three-point starting position recommended in sprint running (Romero-Franco et al., 2017) is not directly transposable to on-ice forward skating. Such subjective data processing may lead to potential approximations or errors and in turn impair inter-trial and inter-rater reliability (Romero-Franco et al., 2017; Stenroth et al., 2020). While ST-TS developed by Stenroth et al. (2020) improved intra-rater reliability, our findings showed that this method, adding a time shift, seems less accurate than the original procedure developed by Romero-Franco et al. (2017).

Several limitations should be kept in mind when interpreting the data from the present study. Firstly, one could note that none of the tested methods includes a direct synchronization between movement kinetics and mechanical variable (e.g., using a trigger signal), which necessarily affect the determination of sprint start and resulting metrics and could be dependant of investigator’s expertise. Future studies may consider to use more than one camera in order to possibly enhance the accuracy of split time assessment. For instance, placing three panning cameras at the start line, 15 and 30 m, in the frontal plane, may allow practitioners to more precisely determine the frame corresponding to the start of the sprint or the time instant at which the skater crosses vertical markers representing split times with parallax correction (Chow, 1993). However, while such setting could improve the measurement accuracy, it would require to synchronize the different devices, which may complicate the data collection and further increase the risk of error. Finally, the sample size precludes any general conclusions and further studies are warranted with larger population, including male or mixed participants.

While easy-to-use, high-speed video-based methods lead to a truncated estimation of mechanical variables compared to radar-derived measures mainly due to subjective determination of the skating sprint start. The implementation of time shift seems to further impair the validity of the measurements. Radar-derived method should be prioritized to accurately determine mechanical variables during on-ice forward skating sprint. Practitioners have to be aware of the limitations of the high-speed video-based methods when implementing them to assess mechanical determinants of the on-ice forward skating sprint.

## Data Availability Statement

The raw data supporting the conclusions of this article will be made available by the authors, without undue reservation.

## Ethics Statement

The studies involving human participants were reviewed and approved by the Ouest IV. Written informed consent to participate in this study was provided by the participants’ legal guardian/next of kin.

## Author Contributions

JP, GG, and FB contributed to conception and design of the study and wrote sections of the manuscript. JP performed the statistical analysis. All authors contributed to the manuscript revision, read, and approved the submitted version.

## Conflict of Interest

The authors declare that the research was conducted in the absence of any commercial or financial relationships that could be construed as a potential conflict of interest.

## Nomenclature

a_h_ Acceleration

*d* Cohen’s *d* scale

D_rf_ Rate of decrease of the ratio of the horizontal component of the ground-reaction force to the corresponding resultant force (%)

F_0_ Maximal theoretical horizontal force relative to body mass (N.kg^–1^)

F_aero_ Aerodynamic friction force (N)

F_h_ Net horizontal antero-posterior ground reaction force (N)

FV Force-velocity relationship

ICC Intra-class correlation coefficients

*m* System mass (kg)

P_h_ Mean net horizontal antero-posterior power output (W)

P_max_ Maximal theoretical horizontal power relative to body mass (W.kg^–1^)

*r* Pearson’s product–moment correlation

RF_max_ Maximal ratio of the horizontal component of the ground-reaction force to the corresponding resultant force (%)

ST Split time method using high-speed video

ST-TS Split time with time shift method using high-speed video

V_0_ Maximal theoretical horizontal velocity (m.s^–1^)

V_h_ Instantaneous horizontal velocity (m.s^–1^)

V_max_ Maximal horizontal velocity (m.s^–1^)

τ Acceleration time constant (m.s^–2^).
